# Psychological determinants of influenza vaccination

**DOI:** 10.1186/s12877-017-0597-y

**Published:** 2017-08-29

**Authors:** Jens-Oliver Bock, André Hajek, Hans-Helmut König

**Affiliations:** Department of Health Economics and Health Services Research, Hamburg Center for Health Economics, University Medical Center Hamburg-Eppendorf, Martinistraße 52, 20246 Hamburg, Germany

**Keywords:** Psychology, Perceived stress, Self-esteem, Influenza vaccination

## Abstract

**Background:**

Previous studies investigated the determinants of individuals’ decision to vaccinate against influenza primarily focusing on social as well as certain proximal determinants, for example, behavioral beliefs. Thus, so far, the analysis of psychological factors as determinants of influenza vaccination was mainly limited to beliefs, attitudes or perceptions that were directly related to influenza vaccination and its perceived impact. However, considering general psychological factors, like general self-efficacy, optimism or subjective well-being, might further enhance the understanding of why certain people vaccinate while others do not. The aim was to investigate the relationship between various general psychological factors and older people’s decision to vaccinate against seasonal flu.

**Methods:**

The data of individuals aged 60 or older (*n* = 5037; in 2014) were used from the Germany Ageing Survey. The data were collected in face-to-face interviews and in self-administered questionnaires. They include questions on the use of influenza vaccination and the psychological factors of optimism, self-efficacy, self-esteem, perceived stress, self-regulation, life satisfaction, and negative affect as well as positive affect. The psychological determinants of regular influenza vaccination were investigated using multiple logistic regressions.

**Results:**

53.2% of all participants were regular users of influenza vaccination. There were significant bivariate correlations of all cited psychological factor with influenza vaccination except for life satisfaction and negative affect. After controlling for numerous potential socio-demographic, morbidity- and lifestyle-related confounders, regular influenza vaccination was still positively associated with lower levels of self-esteem and a higher level of perceived stress.

**Conclusions:**

There are significant associations of general individual psychological constructs with the decision to vaccinate against influenza. Future research might determine the impact of psychological factors on the decision to vaccinate in longitudinal research designs. This might be helpful to understand the causal mechanisms behind this relationship, which could help to design interventions that increase vaccination rates in certain target groups.

**Electronic supplementary material:**

The online version of this article (10.1186/s12877-017-0597-y) contains supplementary material, which is available to authorized users.

## Background

Seasonal flu (influenza) is an infectious disease whose consequences are particularly severe for certain vulnerable groups like older persons, children or pregnant women. According to the World Health Organization (WHO), influenza yearly leads to about 300,000 to 500,000 deaths worldwide [1]. Seasonal flu can be effectively prevented by vaccination. This has been shown for healthy adult persons [2] as well as for the specific risk groups of pregnant women [3], health care workers [4] or older adults [5, 6]. As the virus pathogen changes over time, effective vaccination requires annual repetition [7, 8].

In Germany, no type of vaccination is compulsory. Yet, the German Standing Committee on Vaccination (STIKO) defines recommendations including flu vaccination. Due to Germany’s federal structure, each of its 16 federal states publicly provides recommendations for vaccinations that are based on STIKO’s suggestions. Currently, the STIKO recommends flu vaccination for persons above age 60, for pregnant women, persons suffering from certain chronic conditions, as well as residents of nursing homes [8]. In addition, certain groups at risk that include in particular health care workers, are recommended to vaccinate [8]. General Practitioners (GPs) practices are the usual place where patients get vaccinated for the flu. All sickness funds reimburse the costs for vaccination if it is recommended for a patient. For all other patients, it depends on the statutes of their health insurance whether flu vaccination is free of charge for them.

As flu vaccination is not compulsory but recommended for various groups, many studies have analyzed the determinants of vaccination, especially for these recommendation groups. Thus, for example, concerning the risk group of health care workers, a review of the literature identified 13 studies investigating the determinants of vaccination [9]. This study showed that main predictors investigated so far covered both determinants on the individual level as well as those related to the health care system. For the target group of older people, there are similar reviews [10, 11] that equally focused predominantly on socioeconomic determinants, like age, gender, marital status, educational level, income, etc. Ward and Raude [12] provide a comprehensive framework for understanding influenza vaccination behaviors.

Furthermore, previous studies investigated certain psychological factors as determinants of flu vaccination. For example, a literature review on determinants of uptake of influenza vaccination in pregnant women assessed 21 studies, finding that mainly fear of adverse outcomes as well as doubts about effectiveness of flu vaccination prevent pregnant women from vaccinating against flu [13]. Psychological factors investigated in previous studies were primarily specific health beliefs on flu vaccination – often based on the Health Belief Model – like risk assessment [14], illness-specific perceived self-efficacy [15] or certain emotions related to flu vaccination [16–18]. Thus, so far, the review on psychological factors as determinants of flu vaccination was mainly limited to beliefs, attitudes or perceptions that were *directly related* to flu vaccination and its perceived impact.

Yet considering *general* psychological factors, like general self-efficacy, optimism or subjective well-being, might further enhance the understanding of why certain people vaccinate while others do not. They might play a substantial role as determinants of the use of flu vaccination as they are commonly known to interact in various ways with health behavior [19]. In addition, the decision whether to vaccinate or not is indeed the result of the individual’s direct assessment of the potential harm and benefit of flu vaccination. In sum, we assume that illness-related psychological factors (e.g., illness-specific self-efficacy) are strongly associated with the more general psychological constructs.

Therefore, the aim of this study was to analyze the uptake of flu vaccination with a particular focus on general individual psychological factors. The latter included widely accepted and broadly applied psychological constructs of optimism, self-efficacy, self-esteem, perceived stress, self-regulation, life satisfaction, and affective well-being. This analysis might reveal further determinants of the uptake of flu vaccination, which could be helpful as additional indicators to address persons who are recommended to vaccinate [20]. The determinants of utilization of flu vaccination were investigated in a cross-sectional representative sample of older community-dwelling Germans aged 60 years and older.

We hypothesize that subjective well-being (life satisfaction and affective well-being) as well as optimism is positively associated with the probability of flu vaccination because it has been demonstrated that these factors are associated with health preventive behavior [21, 22]. It has also been shown that self-efficacy is associated with screening behavior [23]. Thus, we hypothesize that general self-efficacy is positively associated with the probability of flu vaccination. Individuals with high self-regulation have a high willingness to make short-term sacrifices (e.g., flu vaccination) in order to achieve long-term goals (e.g., sustaining good health). Consequently, we hypothesize that self-regulation is positively associated with the probability of flu vaccination. Moreover, we hypothesize that self-esteem is positively associated with the probability of flu vaccination because self-esteem is positively linked to body image [24] which in turn is associated with screening behavior [25]. In addition, we hypothesize that stress is negatively associated with the probability of flu vaccination since stress is negatively associated with health-promotion behavior [26].

## Methods

### Sample

The data came from the German Ageing Survey (DEAS). DEAS combined panel samples with a cross sectional sample for the year 2014. In five points in time (1996, 2002, 2008, 2011 and 2014), representative samples for Germany of community-dwelling adults aged 40 years or above were drawn by national probability sampling. The participants were asked for informed consent before taking part in the study. In total, in the year 2014, 10,355 persons were interviewed, with 6003 (response rate: 25%) participants being interviewed for the first time while the remaining 4352 (response rate: 61%) had already participated in a previous round of the DEAS study. Eligible participants provided data on various aspects in interviews based on standardized questionnaires at their homes. *N* = 7750 out of the 10,355 provided information on psychological measures and on whether they regularly used flu vaccination in an additional questionnaire following the interview. The final sample is drawn from these *N* = 7750 persons but restricted to the age group of 60 year and above, thus targeting only individuals for whom regular vaccination is recommended (*n* = 5037). Further details on the 2014 wave of the DEAS study have been published elsewhere [27].

### Variables

#### Outcome: Utilization of flu vaccination

Subsequent to a section of various questions on health care use, data on uptake of flu vaccination were collected as follows: “Doctors often recommend vaccinations and various types of health screening. In the past years, did you regularly get a flu vaccination?” Participants could tick the boxes “yes” or “no”.

#### Socio-demographics and health-related variables

Beyond age in years, gender and dichotomized marital status (married, living together with spouse vs. others (married, living separated from spouse; divorced; widowed; single), we considered individual monthly net equivalence income as well as the region of Germany (West vs. East = region of the former German Democratic Republic). As lifestyle variables, current smoker status (daily, sometimes, not anymore, never been smoker), usual alcohol consumption per week in five categories, and the Body Mass Index (BMI) were considered. The morbidity was assessed using the total number of physical chronic conditions like cardiac and circulatory disorders, diabetes or cancer. In sensitivity analysis, age group dummies were used (allowing for non-linear age effects): 60–69 years, 70–79 years, and ≥80 years. In additional analysis, it was also controlled for employment status (Ref.: employed; retired; other: not employed).

#### Psychological factors

Optimism was assessed using a scale developed by Brandtstädter and Wentura [28]. This 5-items scale measures optimism by four corresponding answers, each ranging from 1 = ‘strongly agree’ to 4 = ‘strongly disagree’. The final score with a range of 1 to 4 is built up as the mean of at least three required valid values. Higher values in the final score represent a higher level of optimism (Cronbach’s alpha = .84). For further details with regard to the psychological factors used in the present study, please see the Additional file 1: Table S1.

Self-efficacy, the belief in the own capacity to reach goals, was measured using a 5-items scale with four levels each [29, 30]. The mean of at least three required valid answers defines the final score, with higher values representing higher levels of self-efficacy. The scale can range from 1 to 4 (Cronbach’s alpha = .84).

Perceived stress was assessed via a 4-items scale by Cohen and colleagues [31]. Each item offers four levels, varying between 0 = ‘never’ and 5 = ‘very often’. Two out of four items must at least be valid so that the overall score can be calculated as the mean of all valid items (Cronbach’s alpha = .70).

Self-esteem, representing the emotional evaluation of one’s own worth, was assessed by the Rosenberg-scale that contains 10 items with four levels each [32]. Participants could 1 = ‘strongly agree’ or, in the complete opposite case, 4 = ‘strongly disagree’ to the 10 questions on self-esteem. The mean of at least three required valid answers defines the final score with higher values representing more self-esteem (Cronbach’s alpha = .84).

We assessed satisfaction with life on the established satisfaction with life scale (SWLS) consisting of five items with five levels each [33]. At least three values must be valid to define the final scale as the mean of all items. This final scale ranges from one to five with higher values corresponding to more satisfaction (Cronbach’s alpha = .86).

Self-regulation, the ability to override short-term desires in order to pursue long-terms goals [34], was measured according to a scale taken from Ziegelmann and Lippke [35] that is based on the concept of selection, optimization, and compensation (SOC) and a corresponding questionnaire [36]. Answers to four items were assigned one to four points each. The mean score of all – but at least two – required valid answers defines the final score that can range one to four with higher values representing a high degree of self-regulation (Cronbach’s alpha = .78).

Positive affect as well as negative affect were assessed based on the Positive and Negative Affect Schedule (PANAS) [37]. Participants assessed their agreement with 20 emotion words by assigning 1 = ‘very slightly‘to 5 = ‘very much’ points each. The mean of the ten positive items defines the final PA scale, whilst the mean of the 10 negative emotions creates the final NA score, both ranging from one to five. For both the negative affect and positive affect scale, at least three valid items are required (PA: Cronbach’s alpha = .87; NA: Cronbach’s alpha = .86).

### Statistical analyses

The differences between regular user and non-user of flu vaccination were analyzed using Student’s t-test for metric variables and Chi^2^ tests for proportions. Multivariate analyses of determinants of flu vaccination included logistic regression models with the regular use (0 = no, 1 = yes) of flu vaccination as dependent variable. Odds ratios (OR) were reported. As various investigated psychological factors tend to be strongly correlated with each other (e.g., self-esteem and optimism: *r* = .61), they were entered separately into the regression analyses. Moreover, they were entered separately in our logistic regression models because our aim was to show that whenever one of these factors is available, one should include it as an explanatory variable. As missing values accounted for less than 2% in each variable (exception: income with 5.5% of missing values), we run complete case analyses in regression models, which is also known as list wise deletion. For all statistical analyses, Stata 14.0 was used and the level of statistical significance was set at 0.05.

## Results

### Sample characteristics

Table 1 shows the sample characteristics by flu vaccination status. The total sample was aged between 60 and 95 with a mean age of 71 years. A slight minority of 48% of all participants was female, roughly two third lived in a Western federal state of Germany, and 71% were married. The average participant was overweight with a BMI of 27 (SD: 4) and earned a monthly net equivalence income of €1870 (SD: €1402). Nearly one-half has never been smoker, 40% were ex-smoker whereas only 9% were currently daily smoker. The participants reported their alcohol consumption to take place ‘daily’ (14%) or ‘never’ (13%) with the remaining 73% drinking between ‘several time a week’ and ‘less than once a month’. 53.2% of all participants were regular user of flu vaccination.

**Table 1:** Sample characteristics, total and by status of flu vaccination *N* = 5037

Variables	Total *N* = 5037 (100%)		Non-user *n* = 2357 (46.8%)		Regular user *n* = 2680 (53.2%)		*p*-value^1^	Missings (%)
	N/Mean	%/SD	N/Mean	%/SD	N/Mean	%/SD		
Gender: Female	2647	47.5%	1120	47.5%	1270	47.4%	0.927	0.0
Age in years	71.2	(7.2)	69.7	(6.9)	72.6	(7.2)	0.000	0.0
Marital status: Others (married, living separated from spouse; divorced; widowed; single) than ‘married and living together with spouse’	1478	29.4%	709	30.2%	769	28.7%	0.262	0.2
Monthly net equivalence income in Euro	1870.3	(1401.6)	2011.2	(1573.3)	1747.7	(1220.0)	0.000	5.5
Region: East Germany	1711	34.0%	525	22.3%	1186	44.3%	0.000	0.0
Body-Mass-Index (BMI)	27.0	(4.4)	26.7	(4.2)	27.4	(4.5)	0.000	1.3
Smoking status: Daily	445	8.9%	257	11.0%	188	7.1%	0.000	1.1
- Yes, sometimes	144	2.9%	81	3.5%	63	2.4%		
- Not anymore	2010	40.3%	933	39.9%	1077	40.7%		
- Never been smoker	2385	47.9%	1065	45.6%	1320	49.9%		
Consumption of alcohol: Daily	690	13.8%	372	15.8%	318	11.9%	0.000	0.4
- Several times a week	1165	23.2%	573	24.4%	592	22.2%		
- Once a week	732	14.6%	347	14.8%	385	14.4%		
- One to three times a month	555	11.1%	265	11.3%	290	10.9%		
- Less frequently	1242	24.8%	522	22.2%	720	27.0%		
- Never	635	12.7%	272	11.6%	363	13.6%		
Number of physical illnesses	3.0	(1.9)	2.6	(1.8)	3.3	(1.9)	0.000	1.7
Life satisfaction (Pavot/Diener 1993) [33]	3.86	(0.70)	3.87	(0.70)	3.85	(0.70)	0.440	1.2
Positive affect (Watson, Clark & Tellegen 1988) [37]	3.53	(0.52)	3.56	(0.53)	3.50	(0.52)	0.000	1.3
Negative affect (Watson, Clark & Tellegen 1988) [37]	2.03	(0.50)	2.02	(0.49)	2.04	(0.50)	0.096	1.3
Optimism (Brandstädter/Wentura 1994) [28]	2.94	(0.55)	2.99	(0.55)	2.90	(0.55)	0.000	0.5
Self-efficacy (Schwarzer/Jerusalem 1995) [30]	3.07	(0.44)	3.10	(0.44)	3.04	(0.44)	0.000	0.6
Self-esteem (Rosenberg 1965) [32]	3.40	(0.39)	3.42	(0.38)	3.37	(0.39)	0.000	0.2
Self-regulation (Freund/Baltes 2002) [36]	3.00	(0.52)	2.99	(0.52)	3.02	(0.51)	0.042	2.4
Perceived stress (Cohen et al. 1983) [31]	2.33	(0.64)	2.27	(0.65)	2.37	(0.63)	0.000	1.9

^1^ *p*-value resulting from Chi^2^ or t-test, respectively; The number of physical illnesses ranged from 0 to 11; Life satisfaction was quantified using the Satisfaction with Life Scale (ranging from 1 to 5, with higher values corresponding to more satisfaction); Positive and negative affect was quantified using the Positive and Negative Affect Schedule (both ranging from 1 to 5, with higher values corresponding to higher positive and negative affect, respectively); Optimism was quantified using a scale developed by Brandtstädter and Wentura (ranging from 1 to 4, with higher values corresponding to higher optimism); Self-efficacy was quantified using a scale developed by Schwarzer and Jerusalem (ranging from 1 to 4, with higher values corresponding to higher self-efficacy); Self-esteem was quantified using the Rosenberg scale (ranging from 1 to 4, with higher values corresponding to higher self-esteem); Self-regulation was quantified using a scale developed by Ziegelmann and Lippke (ranging from 1 to 4, with higher values corresponding to higher self-regulation); Perceived stress was using a scale developed by Cohen and colleagues (ranging from 1 to 5, with higher values corresponding to higher perceived stress)

There were statistically significant differences in most of the described variables according to vaccination status. Thus, the participants with regular flu vaccination were on average about 3 years older than the participants without regular flu vaccination. The regular users were more likely to live in Eastern parts of Germany, had a lower income and a higher BMI. In addition, they suffered on average from 0.7 more chronic conditions. Equally, there were differences in smoking and drinking behavior comparing both groups (i. e., frequency of alcohol consumption and smoking were both negatively associated with odds to vaccinate). Only the gender and marital status did not differ significantly between groups.

Table 1 also shows means and standard deviations of the eight psychological factors by status of flu vaccination. The comparison of the means for user and non-user of flu vaccination shows statistical significant differences in all considered psychological factor except for life satisfaction and negative affect. Users of regular flu shots had lower positive affect, lower optimism, lower self-efficacy, less self-esteem, a higher degree of self-regulation, and more perceived stress.

### Multivariate analysis

Table 2 shows the results of multiple logistic regression models with the regular use of flu vaccination as dependent variable. In contrast to the bivariate analyses, multivariate analyses showed that most psychological factors were not associated with flu vaccination. The OR of six out of the eight psychological factors were found to be not significantly different from 1. This applies to life satisfaction, positive and negative affect, optimism, as well as self-efficacy and self-regulation. Yet, self-esteem was negatively associated with odds to vaccinate (OR: 0.84, CI: 0.71-0.99; *p* < 0.05), while perceived stress was positively associated with odds to vaccinate (OR: 1.16, CI: 1.05-1.29; *p* < 0.01).

**Table 2:** Results of logistic regression model: dependent variable: regular vaccination: 0 = No, 1 = Yes; odds ratios (95% confidence intervals) are displayed

Independent variables	(1)	(2)	(3)	(4)	(5)	(6)	(7)	(8)
Psychological factor included in regression model:	Life satisfaction, (Pavot/Diener 1993) [33]	Positive affect (Watson, Clark & Tellegen 1988) [37]	Negative affect (Watson, Clark & Tellegen 1988) [37]	Optimism (Brandstädter/Wentura 1994) [28]	Self-efficacy (Schwarzer/Jerusalem 1995) [30]	Self-esteem (Rosenberg 1965) [32]	Self-regulation (Freund/Baltes 2002) [36]	Perceived stress (Cohen et al. 1983) [31]
Gender: Female^a^	1.030	1.020	1.010	1.026	1.027	1.034	1.033	1.003
	(0.896 – 1.183)	(0.888 – 1.172)	(0.879 – 1.161)	(0.893 – 1.177)	(0.895 – 1.179)	(0.901 – 1.187)	(0.899 – 1.188)	(0.873 – 1.152)
Age in years	1.063^***^	1.063^***^	1.064^***^	1.061^***^	1.062^***^	1.062^***^	1.063^***^	1.062^***^
	(1.053 – 1.074)	(1.052 – 1.073)	(1.053 – 1.074)	(1.051 – 1.072)	(1.052 – 1.072)	(1.052 – 1.072)	(1.053 – 1.074)	(1.052 – 1.072)
Marital status: Not married^b^	0.797^**^	0.803^**^	0.805^**^	0.794^**^	0.800^**^	0.799^**^	0.801^**^	0.812^**^
	(0.690 – 0.920)	(0.696 – 0.927)	(0.698 – 0.929)	(0.689 – 0.916)	(0.694 – 0.922)	(0.693 – 0.921)	(0.693 – 0.925)	(0.703 – 0.936)
Monthly net equivalence income	1.000	1.000	1.000	1.000	1.000	1.000	1.000	1.000
	(1.000 – 1.000)	(1.000 – 1.000)	(1.000 – 1.000)	(1.000 – 1.000)	(1.000 – 1.000)	(1.000 – 1.000)	(1.000 – 1.000)	(1.000 – 1.000)
Region: East	2.776^***^	2.756^***^	2.783^***^	2.770^***^	2.780^***^	2.783^***^	2.724^***^	2.812^***^
	(2.418 – 3.188)	(2.401 – 3.165)	(2.423 – 3.198)	(2.414 – 3.179)	(2.422 – 3.191)	(2.425 – 3.194)	(2.370 – 3.131)	(2.447 – 3.231)
Body-Mass-Index (BMI)	1.021^**^	1.020^**^	1.021^**^	1.021^**^	1.020^**^	1.020^**^	1.020^*^	1.019^*^
	(1.005 – 1.036)	(1.005 – 1.036)	(1.006 – 1.037)	(1.005 – 1.036)	(1.005 – 1.036)	(1.005 – 1.036)	(1.005 – 1.035)	(1.004 – 1.035)
Smoking status: Yes, sometimes^c^	1.040	1.040	1.042	1.033	1.038	0.998	1.087	1.026
	(0.682 – 1.588)	(0.682 – 1.588)	(0.683 – 1.590)	(0.678 – 1.576)	(0.681 – 1.584)	(0.655 – 1.518)	(0.710 – 1.664)	(0.673 – 1.565)
- Not anymore^c^	1.139	1.134	1.132	1.130	1.132	1.141	1.124	1.195
	(0.897 – 1.445)	(0.893 – 1.439)	(0.892 – 1.438)	(0.891 – 1.433)	(0.893 – 1.435)	(0.900 – 1.447)	(0.884 – 1.430)	(0.941 – 1.518)
- Never been smoker^c^	1.075	1.071	1.069	1.070	1.073	1.081	1.065	1.127
	(0.847 – 1.364)	(0.844 – 1.358)	(0.843 – 1.356)	(0.844 – 1.356)	(0.847 – 1.359)	(0.853 – 1.369)	(0.838 – 1.354)	(0.888 – 1.431)
Consumption of alcohol: several times a week^d^	1.380^**^	1.374^**^	1.376^**^	1.363^**^	1.359^**^	1.369^**^	1.390^**^	1.362^**^
	(1.119 – 1.701)	(1.114 – 1.694)	(1.116 – 1.698)	(1.106 – 1.681)	(1.103 – 1.675)	(1.110 – 1.687)	(1.125 – 1.716)	(1.103 – 1.681)
- Once a week^d^	1.361^*^	1.358^*^	1.360^*^	1.361^*^	1.360^*^	1.369^**^	1.379^**^	1.344^*^
	(1.074 – 1.723)	(1.072 – 1.721)	(1.073 – 1.723)	(1.075 – 1.722)	(1.075 – 1.721)	(1.082 – 1.733)	(1.088 – 1.749)	(1.060 – 1.703)
- One to three times a month4	1.359^*^	1.368^*^	1.366^*^	1.356^*^	1.358^*^	1.346^*^	1.353^*^	1.352^*^
	(1.055 – 1.750)	(1.062 – 1.762)	(1.060 – 1.760)	(1.053 – 1.745)	(1.054 – 1.748)	(1.046 – 1.733)	(1.049 – 1.745)	(1.049 – 1.743)
- Less frequently^d^	1.569^***^	1.566^***^	1.572^***^	1.554^***^	1.573^***^	1.545^***^	1.556^***^	1.500^***^
	(1.262 – 1.952)	(1.259 – 1.949)	(1.264 – 1.957)	(1.250 – 1.932)	(1.265 – 1.955)	(1.243 – 1.920)	(1.249 – 1.938)	(1.205 – 1.868)
- Never^d^	1.479^**^	1.491^**^	1.491^**^	1.484^**^	1.488^**^	1.490^**^	1.576^***^	1.495^**^
	(1.149 – 1.903)	(1.159 – 1.918)	(1.159 – 1.918)	(1.154 – 1.907)	(1.158 – 1.912)	(1.159 – 1.914)	(1.222 – 2.034)	(1.160 – 1.926)
Number of physical illnesses	1.116^***^	1.120^***^	1.111^***^	1.113^***^	1.118^***^	1.111^***^	1.121^***^	1.106^***^
	(1.076 – 1.157)	(1.081 – 1.161)	(1.071 – 1.153)	(1.074 – 1.154)	(1.078 – 1.159)	(1.072 – 1.152)	(1.081 – 1.162)	(1.067 – 1.147)
Psychological factor	0.948	1.014	1.114	0.895^+^	0.893	0.841^*^	1.098	1.160^**^
	(0.863 – 1.042)	(0.895 – 1.149)	(0.975 – 1.272)	(0.794 – 1.008)	(0.771 – 1.035)	(0.712 – 0.994)	(0.971 – 1.241)	(1.048 – 1.285)
Constant	0.00394^***^	0.00320^***^	0.00251^***^	0.00512^***^	0.00501^***^	0.00636^***^	0.00226^***^	0.00254^***^
	(0.00161 – 0.00966)	(0.00118 – 0.00867)	(0.000986 – 0.00641)	(0.00201 – 0.0131)	(0.00190 – 0.0132)	(0.00227 – 0.0178)	(0.000847 – 0.00602)	(0.00104 – 0.00617)
Observations	4525	4522	4521	4557	4553	4567	4477	4500
Pseudo R^2^	0.0898	0.0894	0.0898	0.0900	0.0905	0.0899	0.0902	0.0909

Reference category: ^a^male ^b^married ^c^daily smoker ^d^daily; ^***^ *p* < .001, ^**^ *p* < .01, ^*^ *p* < .05; ^+^ *p* < .10; The number of physical illnesses ranged from 0 to 11; Life satisfaction was quantified using the Satisfaction with Life Scale (ranging from 1 to 5, with higher values corresponding to more satisfaction); Positive and negative affect was quantified using the Positive and Negative Affect Schedule (both ranging from 1 to 5, with higher values corresponding to higher positive and negative affect, respectively); Optimism was quantified using a scale developed by Brandtstädter and Wentura (ranging from 1 to 4, with higher values corresponding to higher optimism); Self-efficacy was quantified using a scale developed by Schwarzer and Jerusalem (ranging from 1 to 4, with higher values corresponding to higher self-efficacy); Self-esteem was quantified using the Rosenberg scale (ranging from 1 to 4, with higher values corresponding to higher self-esteem); Self-regulation was quantified using a scale developed by Ziegelmann and Lippke (ranging from 1 to 4, with higher values corresponding to higher self-regulation); Perceived stress was using a scale developed by Cohen and colleagues (ranging from 1 to 5, with higher values corresponding to higher perceived stress)

In all calculated models, higher age, being married and living together with spouse, living in the state of East Germany, higher BMI as well as higher number of chronic conditions was positively significantly associated with the regular use of flu vaccination. Moreover, drinking alcohol less than daily increased odds to vaccinate. In contrast, there was no significant association of gender, income and smoking status with flu vaccination.

In sensitivity analysis (results not shown, but available upon request), the linear age term was replaced by age group dummies. For example, in the first model (with life satisfaction as explanatory variable), the probability of vaccination increased with age group (ref.: 60–69 years; 70–79 years, OR: 1.50, CI: 1.29-1.74, *p* < .001; ≥ 80 years, OR: 2.41, CI: 1.92-3.02, *p* < .001).

In further sensitivity analysis, employment status was added to the main model (results not shown, but available upon request). The probability of vaccination was positively associated with retirement and other (not employed; ref.: employed). While self-esteem was only marginally significant (*p* = .055), stress remained significant (*p* < .01) after adjustment for employment status.

## Discussion

### Main findings

The aim of this study was to analyze the determinants of a person’s decision to use regular flu vaccination with a particular focus on various general psychological factors such as life satisfaction, optimism, self-efficacy or self-regulation. Using cross-sectional data from a large representative sample of older Germans between 60 and 85 years, we found strong bivariate associations between the use of flu vaccination and the psychological constructs of positive and negative affect, optimism, self-efficacy, self-esteem, and self-regulation, as well as perceived stress. However, in multiple regression models controlling for major confounders including age, gender, marital status, and the number of chronic diseases, only two psychological factors remained determinants of flu vaccination. Lower levels of self-esteem and a higher level of perceived stress were associated with an increased likelihood of getting regular flu shots.

### Possible explanations

Concerning self-esteem, this finding might appear a little counter-intuitive, because it has been shown that self-esteem is a correlate of social support [38], with social support being a predictor of healthy general lifestyles [39, 40]. Consequently, a study even found a causal effect of self-esteem on a healthier lifestyle [41]. Yet, there are important differences to our study. First, outcome measures deviate. In the cited study the combination of lifestyle indicators, which covered nutrition, exercise or substance abuse was the outcome measure, whereas we investigate the specific preventive behavior of flu vaccination. Second, Muhlenkamp and Sayles [41] used a highly specific sample, consisting of *n* = 98 adults living in the same apartment complex, while we used a representative sample in older age of an entire country.

An explanation for our finding might be that persons with high self-esteem have been proven to use more often cognitive strategies to minimize potentially harmful consequences of their individual risky behavior as compared to those with low self-esteem [42]. This makes in particular sense considering the fact that a high self-esteem is not related to one’s own accomplishments or positive experiences [43]. Thus, individuals with high self-esteem tend to ignore disagreeable information, convincing themselves that bad things resulting from their acts cannot happen, which has been shown to increase risky behaviors like drinking alcohol or taking drugs [42].

A higher level of perceived stress was associated with a higher likelihood to vaccinate. A possible explanation of this might be that perceived stress positively correlates with anxiety [44, 45]. A high level of anxiety might correspond to an increased worry about consequences of getting a flu, with the latter known to be associated with an increased probability to vaccinate [16].

The remaining socio-demographic, lifestyle-related and morbidity-related determinants of flu vaccination are comparable to those found in previous studies from Germany [46–48]. Thus, for example, it is well known that vaccination rates are much higher in East Germany due to compulsory vaccination in the former German Democratic Republic. The strong associations of higher age and higher number of chronic conditions with flu vaccination uptake indicate that vaccination rates are indeed somewhat higher in subgroups where vaccination is explicitly recommended by STIKO though vaccination rates are far from 100%.

The results that self-esteem and perceived stress are associated with individual’s decision to the use flu vaccination show that these general psychological factors are important to consider beyond sociodemographic characteristics or concrete assessment and attitude towards flu vaccination. This extends previous analyses to the domain of more general psychological factors and their relationship to a person’s decision to vaccinate against flu.

### Strengths and limitations

A major strength is that our data are derived from a large representative observational study that allows generalizability of the results for the entire German health care context [49]. Measurement of psychological factors was based on established instruments and data on socio-demographics was comprehensive. Yet all data rely on accuracy of participants’ ability and willingness to report correct data, since all of them were self-reported. For example, it might be the case that the share of regular vaccine users was overestimated in the DEAS study due to social desirability bias. In our study, 53.2% of all participants (60 and over) were regular user of flu vaccination. This is supported by the fact that these figures are similar to the figures found in the GEDA study (57% of the individuals aged 60 and over) [50]. However, the figures reported by the GEDA study combine regular and sporadic users. Similar to other large surveys conducted in Germany [51], the response rate was quite low in the DEAS study. The participation rate might be associated with some of our psychological variables (e.g., satisfaction with life). However, it has been shown that selectivity effects are rather low in the DEAS study [52]. Furthermore, this is a cross-sectional study, with all of its inherent limitations. In addition, future studies are needed to clarify whether there is an intention-action gap [53, 54] in the association between psychological factors and flu vaccination.

## Conclusions

Previous literature on individual determinants of flu vaccination focused – beyond socio-demographics – in particular on psychologic constructs that were directly linked to the assessment of specific aspects of flu vaccination. This covered, for example, the assessments of the risks and the potential benefits from vaccination. As there is ample evidence on the influence of these psychological assessments on the decision to vaccinate, we adopted a broader perspective of psychological factors, using general well-established psychological constructs in order to potentially better understand the use of regular flu vaccination. Beyond strong bivariate correlations of many psychological factors with flu vaccination, self-esteem and perceived stress were associated in multiple regression models with flu vaccination. This knowledge might be helpful to address target groups in order to increase vaccination rates, though the practical importance might be limited because effect sizes were rather low.

Future research might determine the impact of psychological factors on the decision to vaccinate in longitudinal research designs. This might be helpful to understand the causal mechanisms behind this relationship, which could help to design interventions that increase vaccination rates in certain target groups. Both supply and demand interventions might be fruitful to increase vaccination rates [55].

## Additional file

Additional file 1: Psychological factors (items and explanations). (DOCX 16 kb)

## Abbreviations

BMI: Body mass index
DEAS: German Ageing Survey
GP: General practitioner
OR: Odds ratio
PANAS: Positive and negative affect schedule
SD: Standard deviation
STIKO: German standing committee on vaccination
WHO: World Health Organization
