# Supplementary material for: Psychological determinants of influenza vaccination
Source: BMC Geriatr. 2017 Aug 29;17:194. doi: 10.1186/s12877-017-0597-y (PMC5576319; doi:10.1186/s12877-017-0597-y)
Supplement: Additional file 1: — Psychological factors (items and explanations). (DOCX 16 kb) [file 12877_2017_597_MOESM1_ESM.docx]

Additional file 1: Psychological factors (items and explanations)

| Psychological factors | Items and explanations |
| --- | --- |
| Life satisfaction (SWLS, Pavot & Diener, 1993) | 1. In most ways my life is close to my ideal. 2. The conditions of my life are excellent. 3. I am satisfied with my life. 4. So far I have gotten the important things I want in life. 5. If I could live my life over, I would change almost nothing.   Scale represents the mean of at least 3 required valid items, all items have been recoded.  1 = strongly agree  2 = agree  3 = neither agree nor disagree  4 = disagree  5 = strongly disagree  High values represent high life satisfaction. |
| Negative affect (PANAS, Watson et al., 1988) | In the following you will find a number of words that describe different feelings and emotions. Please indicate to what extent you have felt this way during the past few months.   1. Distressed 2. Upset 3. Guilty 4. Scared 5. Hostile 6. Irritable 7. Ashamed 8. Nervous 9. Jittery 10. Afraid   Scale represents the mean of at least 3 required valid items.  1 = very slightly or not at all  2 = a little  3 = moderately  4 = quite a bit  5 = extremely  High values on the NA scale represent high frequency of negative emotions. |
| Positive affect (PANAS, Watson et al., 1988) | In the following you will find a number of words that describe different feelings and emotions. Please indicate to what extent you have felt this way during the past few months.   1. Enthusiastic 2. Excited 3. Strong 4. Interested 5. Proud 6. Alert 7. Inspired 8. Determined 9. Attentive 10. Active   Scale represents the mean of at least 3 required valid items.  1 = very slightly or not at all  2 = a little  3 = moderately  4 = quite a bit  5 = extremely  High values on the PA scale represent high frequency of positive emotions. |
| Optimism (Brandtstädter & Wentura, 1994) | 1. I am looking forward to the life ahead of me. (*) 2. For me the future is full of hope. (*) 3. Thinking about my future makes me worry. 4. I look to the future with confidence. (*) 5. The future holds a lot of good in store for me. (*)   Scale represents the mean of at least 3 required valid items, 4 items have been recoded.  1 = strongly agree  2 = agree  3 = disagree  4 = strongly disagree  High values represent high optimism. |
| Self-efficacy (Schwarzer & Jerusalem, 1999) | 1. It is easy for me to stick to my aims and accomplish my goals. 2. I can usually handle whatever comes my way. 3. I can solve most problems if I invest the necessary effort. 4. If I am in trouble, I can usually think of a solution. 5. When I am confronted with a problem, I can usually find several solutions   Scale represents the mean of at least 3 required valid items, all items have been recoded.  1 = strongly agree  2 = agree  3 = disagree  4 = strongly disagree  High values represent high self-efficacy. |
| Self-esteem (Rosenberg, 1965) | 1. On the whole, I am satisfied with myself. (*) 2. I am able to do things as well as most other people. (*) 3. All in all, I am inclined to feel that I am a failure. 4. I feel that I have a number of good qualities. (*) 5. I certainly feel useless at times. 6. I feel that I‘m a person of worth, at least on an equal plane with others. (*) 7. I feel I do not have much to be proud of. 8. I take a positive attitude toward myself. (*) 9. I wish I could have more respect for myself. 10. At times I think I am no good at all.   Scale represents the mean of at least 3 required valid items, 5 items have been recoded.  1 = strongly agree  2 = agree  3 = disagree  4 = strongly disagree  High values represent high self-esteem. |
| Self-regulation (Freund & Baltes, 2002; Ziegelmann & Lippke, 2006) | 1. I do everything I can to realize my plans. (*) 2. I have set my goals clearly and stick to them. (*) 3. When it becomes harder for me to get the same results, I keep trying harder until I can do it as well as before. (*) 4. When I can’t do something important the way I did before,   I look for a new goal. (*)  Scale represents the mean of at least 2 required valid items, all items have been recoded.  1 = strongly agree  2 = agree  3 = disagree  4 = strongly disagree  High values represent high self-regulation. |
| Stress (Cohen et al., 1983) | 1. In the last month, how often have you felt that you were unable to control the important things in your life? 2. In the last month, how often have you felt confident about your ability to handle your personal problems? (*) 3. In the last month, how often have you felt that things were going your way? (*) 4. In the last month, how often have you felt difficulties were piling up so high that you could not overcome them?   Scale represents the mean of at least 2 required valid items, 2 items have been recoded.  1 = never  2 = seldom  3 = sometimes  4 = often  5 = very often  High values represent high perceived stress. |

Legend: Items with asterisk have been recoded.
